# Selective Internal Radiation Therapy (SIRT) with yttrium-90 resin microspheres plus standard systemic chemotherapy regimen of FOLFOX versus FOLFOX alone as first-line treatment of non-resectable liver metastases from colorectal cancer: the SIRFLOX study

**DOI:** 10.1186/1471-2407-14-897

**Published:** 2014-12-01

**Authors:** Peter Gibbs, Val Gebski, Mark Van Buskirk, Kenneth Thurston, David N Cade, Guy A Van Hazel, SIRFLOX Study Group

**Affiliations:** Royal Melbourne Hospital, Melbourne, Victoria Australia; NHMRC Clinical Trials Centre, Camperdown, New South Wales Australia; Data Reduction LLC, Chester, New Jersey USA; Sirtex Medical Limited, North Sydney, New South Wales Australia; University of Western Australia, Perth, Western Australia Australia; Walter and Eliza Hall Institute Medical Research, Melbourne, Australia

**Keywords:** Metastatic colorectal cancer, Liver metastases, Systemic chemotherapy, SIR-Spheres® microspheres, Selective Internal Radiation Therapy (SIRT)

## Abstract

**Background:**

In colorectal cancer (CRC), unresectable liver metastases are linked to poor prognosis. Systemic chemotherapy with regimens such as FOLFOX (combination of infusional 5-fluorouracil, leucovorin and oxaliplatin) is the standard first-line treatment. The SIRFLOX trial was designed to assess the efficacy and safety of combining FOLFOX-based chemotherapy with Selective Internal Radiation Therapy (SIRT or radioembolisation) using yttrium-90 resin microspheres (SIR-Spheres®; Sirtex Medical Limited, North Sydney, Australia).

**Methods/Design:**

SIRFLOX is a randomised, multicentre trial of mFOLFOX6 chemotherapy ± SIRT as first-line treatment of patients with liver-only or liver-predominant metastatic CRC (mCRC). The trial aims to recruit adult chemotherapy-naïve patients with proven liver metastases with or without limited extra-hepatic disease, a life expectancy of ≥3 months and a WHO performance status of 0–1. Patients will be randomised to receive either mFOLFOX6 or SIRT + mFOLFOX6 (with a reduced dose of oxaliplatin in cycles 1–3 following SIRT). Patients in both arms can receive bevacizumab at investigator discretion. Protocol chemotherapy will continue until there is unacceptable toxicity, evidence of tumour progression, complete surgical resection or ablation of cancerous lesions, or the patient requests an end to treatment. The primary endpoint of the SIRFLOX trial is progression-free survival (PFS). Secondary endpoints include: PFS in the liver; tumour response rate (liver and any site); site of tumour progression; health-related quality of life; toxicity and safety; liver resection rate; and overall survival. Assuming an increase in the median PFS from 9.4 months to 12.5 months with the addition of SIRT to mFOLFOX6, recruiting ≥450 patients will be sufficient for 80% power and 95% confidence.

**Discussion:**

The SIRFLOX trial will establish the potential role of SIRT + standard systemic chemotherapy in the first-line management of mCRC with non-resectable liver metastases.

**Trial registration:**

SIRFLOX ClinicalTrials.gov identifier: NCT00724503. Registered 25 July 2008.

**Electronic supplementary material:**

The online version of this article (doi:10.1186/1471-2407-14-897) contains supplementary material, which is available to authorized users.

## Background

In colorectal cancer (CRC), liver metastases are linked to poor prognosis – death and recurrence are frequently attributable to liver metastases [1–3]. Surgical resection of CRC liver metastases can result in cure, and produces 5-year survival of 27-39% and 10-year survival of 12-36% [2, 4, 5], as opposed to median survival of approximately 9 months if untreated [6]. However, only 10-20% of patients with liver metastases from CRC are candidates for such surgery [7–10], and intra-hepatic and extra-hepatic relapse after liver resection is common [11].

Systemic chemotherapy is, therefore, used as first-line treatment in patients with non-resectable liver metastases [12–17], and in some cases can sufficiently down-size the tumour burden in patients with previously inoperable liver metastases so that they may be converted to candidates for potentially curative resection [18, 19]. Internationally accepted first-line chemotherapy regimens for patients with mCRC include FOLFOX (combination of bolus and infusional 5-fluorouracil [5-FU], leucovorin [LV] and oxaliplatin) and FOLFIRI (combination of bolus and infusional 5-FU, LV and irinotecan). These regimens provide median survival times of 16–20 months [15, 20], and the addition of biologic agents, such as bevacizumab and cetuximab, to chemotherapy regimens may enhance progression-free survival (PFS) and overall survival (OS) [17, 21].

Selective Internal Radiation Therapy (SIRT or radioembolisation) is an innovative radiation therapy for mCRC, which involves the delivery of SIR-Spheres® (Sirtex Medical Limited, North Sydney, Australia), that contain the β-emitter yttrium-90, into the arterial supply of the liver. These resin microspheres are delivered via a trans-femoral hepatic artery catheter. In a randomised Phase II trial, treatment of mCRC with SIRT plus first-line 5-FU/LV chemotherapy resulted in a longer time-to-progression (18.6 months) compared with 5-FU/LV chemotherapy alone (3.6 months) [22]. A subsequent Phase I clinical trial demonstrated that SIRT combined with FOLFOX4 systemic chemotherapy had acceptable tolerability [23]. In this trial of 20 patients with non-resectable liver metastases from CRC, SIRT was administered on the third or fourth day of the first cycle of first-line chemotherapy. The dose-limiting toxicity was grade 3/4 neutropenia, and the authors suggested the maximum tolerated dose of oxaliplatin was 60 mg/m^2^ for the first three cycles, with full-dose FOLFOX4 thereafter [23]. Although the primary endpoint of this study was toxicity, the objective response rate according to Response Evaluation Criteria In Solid Tumours (RECIST) was 90%, two patients (10%) were down-staged to undergo hepatic resection and median PFS was 9.3 months [23].

These results suggest that the combination of SIRT and FOLFOX systemic chemotherapy warrants further investigation. Consequently, two open-label, randomised, controlled Phase III trials of mFOLFOX6 ± SIRT as first-line treatment of patients with liver-only or liver-predominant mCRC were designed with virtually identical protocols (SIRFLOX and FOXFIRE). The primary endpoint of SIRFLOX is a comparison between treatment arms of PFS.

## Methods/Design

The SIRFLOX study will be conducted in accordance with the Declaration of Helsinki, and approval has been obtained from the relevant ethics committees for each participating centre (see Additional file 1 for a list). When the results of the study are reported, CONSORT guidelines will be adhered to.

### Eligible population

The inclusion and exclusion criteria for the SIRFLOX study is summarised in Table 1.

**Table 1:** **Patient eligibility criteria for SIRFLOX study**

Inclusion criteria	Exclusion criteria
● Written informed consent provided.	● Evidence of ascites, cirrhosis, portal hypertension, main portal venous tumour involvement or main portal venous thrombosis.
● Aged ≥18 years with histologically confirmed adenocarcinoma of the colon or rectum (with or without the primary tumour *in situ*).	● Previous radiation therapy to the upper abdomen.
● Proven liver metastases.	● Non-malignant disease that renders patients unsuitable for the study treatment.
● WHO performance status of 0–1.	● Grade >1 peripheral neuropathy (NCI-CTCv3).
● Life expectancy of ≥3 months.	● Previous dose-limiting toxicity associated with adjuvant 5-FU or oxaliplatin chemotherapy.
● Patients with additional limited extra-hepatic metastases in the lung or lymph nodes (fewer than 5 nodules ≤1 cm diameter or a single nodule ≤1.7 cm diameter in the lung, and lymph node involvement in a single anatomical area <2 cm diameter) with the aim of these patients being <40% of the total number of patients recruited (but not being excluded even if they account for more than this proportion).	● Pregnancy or breast-feeding.
● Chemotherapy-naïve for mCRC, but previous adjuvant systemic chemotherapy for primary CRC or neoadjuvant chemo-radiotherapy to the pelvis more than 6 months before recruitment are permitted.	● Current or history of cancer other than adequately treated non-melanoma skin cancer or carcinoma *in situ* of the cervix.
● Deemed suitable for either treatment regimen by the investigator.	● Allergy to non-ionic contrast agents.
● Adequate haematological, renal and hepatic function.	
● Using an acceptable method of contraception.	

### Overview of study design

**Figure 1:**
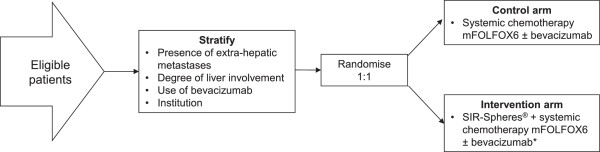
**Overview of SIRFLOX study design.** Figure footnote: * Bevacizumab, at the investigator’s discretion, but not until cycle 4.

SIRFLOX is a randomised, multicentre study of mFOLFOX6 ± SIRT as first-line treatment of patients with inoperable liver-only or liver-predominant mCRC. In SIRFLOX, the aim will be to recruit a minimum of 450 patients at a minimum of 35 sites in Australia, Europe, Israel, New Zealand and USA. Eligible patients are randomised 1:1 to receive either systemic chemotherapy with 5-FU/LV + oxaliplatin (FOLFOX; control arm) or single-session whole liver SIRT + systemic chemotherapy with 5-FU/LV + oxaliplatin (FOLFOX; intervention arm) (Figure 1). All patients may also receive bevacizumab at the investigator’s discretion.

### Randomisation and stratification

When a patient is eligible to participate in the study, randomisation, using the method of minimisation, will be performed using a centralised Study Randomisation Centre at the NHMRC Clinical Trial Centre at the University of Sydney. Treatment will be allocated randomly using the following stratification parameters: liver-only versus extra-hepatic metastases (at least 60% of recruited patients will have liver-only metastases); the extent of tumour involvement of the liver (classed as ≤25% or >25% tumour involvement determined by CT scan, and based upon the tumour involvement groupings used by Gray et al. [24]); planned use of bevacizumab with chemotherapy; and investigational centre.

### Protocol treatment

**Figure 2:**
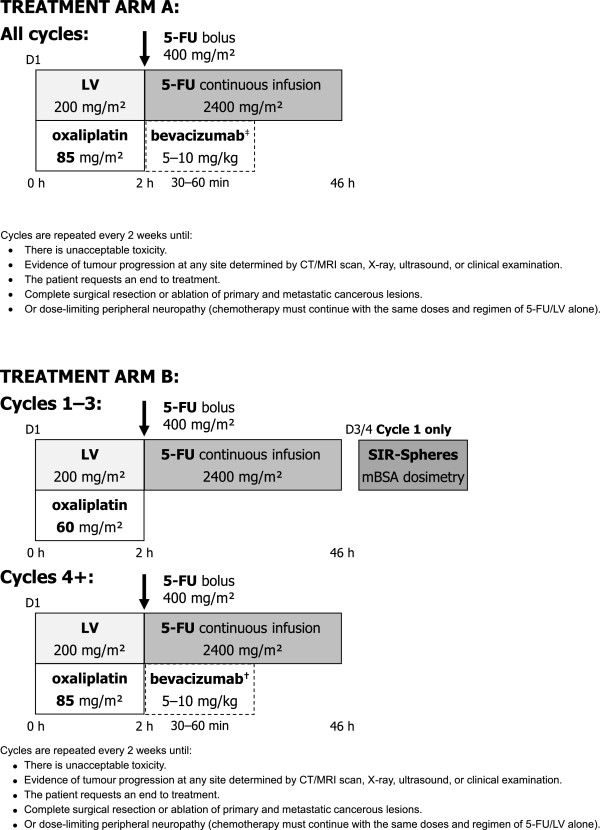
**Treatment received as part of the SIRFLOX study.** Figure footnote: ^‡^Bevacizumab can be added at the investigator’s discretion starting at cycle 1 (or according to the institutional protocol). ^†^Bevacizumab can be added at the investigator’s discretion but should not be initiated until cycle 4 (or according to the institutional protocol). If leucovorin is not available, levofolinic acid can be used at a dose of 100 mg/m^2^. If a centre considers leucovorin 400 mg/m^2^ the standard dose, then this will be allowed at the investigator’s discretion. LV = Leucovorin; 5-FU =5-Fluorouracil.

Systemic chemotherapy must start within 28 days of randomisation. Treatment cycles are described in Figure 2. All patients will be monitored until death or for a minimum of 5 years. Patients randomised to the intervention arm will require a hepatic angiogram and a liver-to-lung shunt study before the SIRT procedure to determine their suitability to receive this treatment. The prescribed activity of SIR-Spheres will be determined from the patient’s body surface area (BSA), the percentage tumour involvement, and the magnitude of liver-to-lung shunting (see Table 2).

**Table 2:** **Administered dose calculator used in the SIRFLOX study**

	Percentage tumour involvement															
BSA	0-5	6-10	11-15	16-20	21-25	26-30	31-35	36-40	41-45	46-50	51-55	56-60	61-65	66-70	71-75	76-80
	**0-10% lung breakthrough**															
1.30-1.35	0.7	0.8	1.0	1.2	1.3	1.4	1.5	1.5	1.5	1.5	1.4	1.3	1.3	1.2	1.1	1.1
1.36-1.40	0.7	0.9	1.0	1.2	1.3	1.4	1.5	1.5	1.5	1.5	1.5	1.4	1.3	1.2	1.2	1.1
1.41-1.45	0.7	0.9	1.1	1.2	1.4	1.5	1.6	1.6	1.6	1.6	1.5	1.4	1.4	1.3	1.2	1.2
1.46-1.50	0.8	0.9	1.1	1.3	1.4	1.5	1.6	1.7	1.7	1.6	1.6	1.5	1.4	1.3	1.3	1.2
1.51-1.55	0.8	1.0	1.1	1.3	1.5	1.6	1.7	1.7	1.7	1.7	1.6	1.5	1.5	1.4	1.3	1.2
1.56-1.60	0.8	1.0	1.2	1.4	1.5	1.6	1.7	1.8	1.8	1.7	1.7	1.6	1.5	1.4	1.3	1.3
1.61-1.65	0.8	1.0	1.2	1.4	1.6	1.7	1.8	1.8	1.8	1.8	1.7	1.6	1.6	1.5	1.4	1.3
1.66-1.70	0.9	1.1	1.3	1.4	1.6	1.7	1.8	1.9	1.9	1.8	1.8	1.7	1.6	1.5	1.4	1.4
1.71-1.75	0.9	1.1	1.3	1.5	1.7	1.8	1.9	1.9	1.9	1.9	1.8	1.7	1.6	1.5	1.5	1.4
1.76-1.80	0.9	1.1	1.3	1.5	1.7	1.8	1.9	2.0	2.0	1.9	1.9	1.8	1.7	1.6	1.5	1.4
1.81-1.85	0.9	1.1	1.4	1.6	1.8	1.9	2.0	2.0	2.0	2.0	1.9	1.8	1.7	1.6	1.5	1.5
1.86-1.90	1.0	1.2	1.4	1.6	1.8	1.9	2.0	2.1	2.1	2.1	2.0	1.9	1.8	1.7	1.6	1.5
1.91-1.95	1.0	1.2	1.4	1.7	1.9	2.0	2.1	2.1	2.1	2.1	2.0	1.9	1.8	1.7	1.6	1.6
1.96-2.00	1.0	1.2	1.5	1.7	1.9	2.1	2.2	2.2	2.2	2.2	2.1	2.0	1.9	1.8	1.7	1.6
2.01-2.05	1.0	1.3	1.5	1.7	1.9	2.1	2.2	2.3	2.3	2.2	2.1	2.0	1.9	1.8	1.7	1.6
2.06-2.10	1.1	1.3	1.6	1.8	2.0	2.2	2.3	2.3	2.3	2.3	2.2	2.1	2.0	1.9	1.8	1.7
2.11-2.15	1.1	1.3	1.6	1.8	2.0	2.2	2.3	2.4	2.4	2.3	2.2	2.1	2.0	1.9	1.8	1.7
2.16-2.20	1.1	1.4	1.6	1.9	2.1	2.3	2.4	2.4	2.4	2.4	2.3	2.2	2.1	1.9	1.8	1.8
2.21-2.25	1.1	1.4	1.7	1.9	2.1	2.3	2.4	2.5	2.5	2.4	2.3	2.2	2.1	2.0	1.9	1.8
2.26-2.30	1.2	1.4	1.7	2.0	2.2	2.4	2.5	2.5	2.5	2.5	2.4	2.3	2.2	2.0	1.9	1.8
2.31-2.35	1.2	1.5	1.7	2.0	2.2	2.4	2.5	2.6	2.6	2.5	2.5	2.3	2.2	2.1	2.0	1.9
2.36-2.40	1.2	1.5	1.8	2.0	2.3	2.5	2.6	2.6	2.6	2.6	2.5	2.4	2.3	2.1	2.0	1.9
2.41-2.45	1.2	1.5	1.8	2.1	2.3	2.5	2.6	2.7	2.7	2.6	2.6	2.4	2.3	2.2	2.0	1.9
2.46-2.50	1.3	1.5	1.8	2.1	2.4	2.6	2.7	2.8	2.8	2.7	2.6	2.5	2.4	2.2	2.1	2.0
	**11-15% lung breakthrough**															
1.30-1.35	0.7	0.8	1.0	1.2	1.3	1.4	1.5	1.5	1.5	1.5	1.4	1.3	1.3	1.2	1.1	1.1
1.36-1.40	0.7	0.9	1.0	1.2	1.3	1.4	1.5	1.5	1.5	1.5	1.5	1.4	1.3	1.2	1.2	1.1
1.41-1.45	0.7	0.9	1.1	1.2	1.4	1.5	1.6	1.6	1.6	1.6	1.5	1.4	1.4	1.3	1.2	1.2
1.46-1.50	0.8	0.9	1.1	1.3	1.4	1.5	1.6	1.7	1.7	1.6	1.6	1.5	1.4	1.3	1.3	1.2
1.51-1.55	0.8	1.0	1.1	1.3	1.5	1.6	1.7	1.7	1.7	1.7	1.6	1.5	1.5	1.4	1.3	1.2
1.56-1.60	0.8	1.0	1.2	1.4	1.5	1.6	1.7	1.8	1.8	1.7	1.7	1.6	1.5	1.4	1.3	1.3
1.61-1.65	0.8	1.0	1.2	1.4	1.6	1.7	1.8	1.8	1.8	1.8	1.7	1.6	1.6	1.5	1.4	1.3
1.66-1.70	0.9	1.1	1.3	1.4	1.6	1.7	1.8	1.9	1.9	1.8	1.8	1.7	1.6	1.5	1.4	1.4
1.71-1.75	0.9	1.1	1.3	1.5	1.7	1.8	1.9	1.9	1.9	1.9	1.8	1.7	1.6	1.5	1.5	1.4
1.76-1.80	0.9	1.1	1.3	1.5	1.7	1.8	1.9	2.0	2.0	1.9	1.9	1.8	1.7	1.6	1.5	1.4
1.81-1.85	0.9	1.1	1.4	1.6	1.8	1.9	2.0	2.0	2.0	2.0	1.9	1.8	1.7	1.6	1.5	1.5
1.86-1.90	1.0	1.2	1.4	1.6	1.8	1.9	2.0	2.0	2.0	2.0	2.0	1.9	1.8	1.7	1.6	1.5
1.91-1.95	1.0	1.2	1.4	1.7	1.9	2.0	2.0	2.0	2.0	2.0	2.0	1.9	1.8	1.7	1.6	1.6
1.96-2.00	1.0	1.2	1.5	1.7	1.9	2.0	2.0	2.0	2.0	2.0	2.0	2.0	1.9	1.8	1.7	1.6
2.01-2.05	1.0	1.3	1.5	1.7	1.9	2.0	2.0	2.0	2.0	2.0	2.0	2.0	1.9	1.8	1.7	1.6
2.06-2.10	1.1	1.3	1.6	1.8	2.0	2.0	2.0	2.0	2.0	2.0	2.0	2.0	2.0	1.9	1.8	1.7
2.11-2.15	1.1	1.3	1.6	1.8	2.0	2.0	2.0	2.0	2.0	2.0	2.0	2.0	2.0	1.9	1.8	1.7
2.16-2.20	1.1	1.4	1.6	1.9	2.0	2.0	2.0	2.0	2.0	2.0	2.0	2.0	2.0	1.9	1.8	1.8
2.21-2.25	1.1	1.4	1.7	1.9	2.0	2.0	2.0	2.0	2.0	2.0	2.0	2.0	2.0	2.0	1.9	1.8
2.26-2.30	1.2	1.4	1.7	2.0	2.0	2.0	2.0	2.0	2.0	2.0	2.0	2.0	2.0	2.0	1.9	1.8
2.31-2.35	1.2	1.5	1.7	2.0	2.0	2.0	2.0	2.0	2.0	2.0	2.0	2.0	2.0	2.0	2.0	1.9
2.36-2.40	1.2	1.5	1.8	2.0	2.0	2.0	2.0	2.0	2.0	2.0	2.0	2.0	2.0	2.0	2.0	1.9
2.41-2.45	1.2	1.5	1.8	2.0	2.0	2.0	2.0	2.0	2.0	2.0	2.0	2.0	2.0	2.0	2.0	1.9
2.46-2.50	1.3	1.5	1.8	2.0	2.0	2.0	2.0	2.0	2.0	2.0	2.0	2.0	2.0	2.0	2.0	2.0
	**16-20% lung breakthrough**															
1.30-1.35	0.7	0.8	1.0	1.2	1.3	1.4	1.5	1.5	1.5	1.5	1.4	1.3	1.3	1.2	1.1	1.1
1.36-1.40	0.7	0.9	1.0	1.2	1.3	1.4	1.5	1.5	1.5	1.5	1.5	1.4	1.3	1.2	1.2	1.1
1.41-1.45	0.7	0.9	1.1	1.2	1.4	1.5	1.5	1.5	1.5	1.5	1.5	1.4	1.4	1.3	1.2	1.2
1.46-1.50	0.8	0.9	1.1	1.3	1.4	1.5	1.5	1.5	1.5	1.5	1.5	1.5	1.4	1.3	1.3	1.2
1.51-1.55	0.8	1.0	1.1	1.3	1.5	1.5	1.5	1.5	1.5	1.5	1.5	1.5	1.5	1.4	1.3	1.2
1.56-1.60	0.8	1.0	1.2	1.4	1.5	1.5	1.5	1.5	1.5	1.5	1.5	1.5	1.5	1.4	1.3	1.3
1.61-1.65	0.8	1.0	1.2	1.4	1.5	1.5	1.5	1.5	1.5	1.5	1.5	1.5	1.5	1.5	1.4	1.3
1.66-1.70	0.9	1.1	1.3	1.4	1.5	1.5	1.5	1.5	1.5	1.5	1.5	1.5	1.5	1.5	1.4	1.4
1.71-1.75	0.9	1.1	1.3	1.5	1.5	1.5	1.5	1.5	1.5	1.5	1.5	1.5	1.5	1.5	1.5	1.4
1.76-1.80	0.9	1.1	1.3	1.5	1.5	1.5	1.5	1.5	1.5	1.5	1.5	1.5	1.5	1.5	1.5	1.4
1.81-1.85	0.9	1.1	1.4	1.5	1.5	1.5	1.5	1.5	1.5	1.5	1.5	1.5	1.5	1.5	1.5	1.5
1.86-1.90	1.0	1.2	1.4	1.5	1.5	1.5	1.5	1.5	1.5	1.5	1.5	1.5	1.5	1.5	1.5	1.5
1.91-1.95	1.0	1.2	1.4	1.5	1.5	1.5	1.5	1.5	1.5	1.5	1.5	1.5	1.5	1.5	1.5	1.5
1.96-2.00	1.0	1.2	1.5	1.5	1.5	1.5	1.5	1.5	1.5	1.5	1.5	1.5	1.5	1.5	1.5	1.5
2.01-2.05	1.0	1.3	1.5	1.5	1.5	1.5	1.5	1.5	1.5	1.5	1.5	1.5	1.5	1.5	1.5	1.5
2.06-2.10	1.1	1.3	1.5	1.5	1.5	1.5	1.5	1.5	1.5	1.5	1.5	1.5	1.5	1.5	1.5	1.5
2.11-2.15	1.1	1.3	1.5	1.5	1.5	1.5	1.5	1.5	1.5	1.5	1.5	1.5	1.5	1.5	1.5	1.5
2.16-2.20	1.1	1.4	1.5	1.5	1.5	1.5	1.5	1.5	1.5	1.5	1.5	1.5	1.5	1.5	1.5	1.5
2.21-2.25	1.1	1.4	1.5	1.5	1.5	1.5	1.5	1.5	1.5	1.5	1.5	1.5	1.5	1.5	1.5	1.5
2.26-2.30	1.2	1.4	1.5	1.5	1.5	1.5	1.5	1.5	1.5	1.5	1.5	1.5	1.5	1.5	1.5	1.5
2.31-2.35	1.2	1.5	1.5	1.5	1.5	1.5	1.5	1.5	1.5	1.5	1.5	1.5	1.5	1.5	1.5	1.5
2.36-2.40	1.2	1.5	1.5	1.5	1.5	1.5	1.5	1.5	1.5	1.5	1.5	1.5	1.5	1.5	1.5	1.5
2.41-2.45	1.2	1.5	1.5	1.5	1.5	1.5	1.5	1.5	1.5	1.5	1.5	1.5	1.5	1.5	1.5	1.5
2.46-2.50	1.3	1.5	1.5	1.5	1.5	1.5	1.5	1.5	1.5	1.5	1.5	1.5	1.5	1.5	1.5	1.5

Dose values are given in GBq.

*BSA* = Body surface area.

The dose of bevacizumab administered in the study will be according to standard institutional protocols (usually 5–10 mg/kg) and should be infused on the first day of each chemotherapy cycle, commencing with cycle 1 in the control arm. In the intervention arm, bevacizumab should be withheld until at least cycle 4 to mitigate the risk of additive toxicity should the non-targeted delivery of SIR-Spheres to the gastrointestinal tract occur. If non-targeted delivery is suspected, then gastroduodenoscopy will be undertaken before the initiation of bevacizumab therapy. If gastroduodenoscopy reveals an ulcer with biopsy-proven microspheres present, bevacizumab will be withheld until resolution of the ulcer.

In both arms, if following treatment response the patient is deemed a candidate for surgical resection, and the patient undergoes surgical resection and/or complete ablation of their primary and metastatic cancer, adjuvant cycles of protocol mFOLFOX6 ± bevacizumab will be continued for a minimum of 12 cycles (including pre-operative cycles).

### Outcome measures

The primary outcome measure of the SIRFLOX study is a comparison between treatment arms of PFS. Secondary outcomes will include: PFS in the liver; OS; tumour response rate (liver and any site); health-related quality of life (HRQoL); toxicity and safety; and liver resection rate.

### Outcome definitions

OS is defined as the time from the date of randomisation to death from any cause. Patients lost to follow-up, withdrawn, or alive at study completion will be censored at the last date that the patient is known to be alive. PFS is defined as the time from the date of randomisation to confirmation of disease progression at any site (RECIST version 1.0 guidelines [25]) or death from any cause if this occurs before disease progression is documented. Patients who change treatment for reasons other than progression (other than patients who are deemed suitable for surgery) will be censored at change of treatment. PFS and tumour response rate will be determined from serial CT scans using RECIST version 1.0 criteria. Centralised assessment of CT scans and tumour response will be conducted by two independent, board-certified radiologists. Cases of disagreement in the judgment of PFS by the two readers will be adjudicated by a medical oncologist based on both radiological and clinical criteria. The PFS based upon the centralised review constitutes the primary endpoint.

HRQoL will be measured using two questionnaires, the EQ-5D and the EORTC QLQ-30. Adverse events (AEs) and Serious Adverse Events (SAEs) will be collected in accordance with ISO14155 and the International Conference on Harmonisation (ICH) guidelines, and will be rated according to Common Terminology Criteria for Adverse Events (CTCAE) v3.0 and the relationship to protocol therapy will be rated as none, unlikely, possible or probable.

After 360 patients (80% of the intended sample size) have completed 15 months of follow-up, at least 300 progressions are expected. The actual pooled event rate will be assessed at this time by an independent data monitoring and safety monitoring committee (IDMC) to determine if the study should continue as planned.

### Assessment procedures and timing

All patients will be assessed by the criteria summarised in Table 3. Additional non-study assessments are permitted at the discretion of the treating investigator.

**Table 3:** **SIRFLOX study assessment schedule**

	Screening	During chemotherapy			Post-progression follow-up
Evaluation/examination	(≤28 d before randomisation)	Day 1	Day 3 or 4 of cycle 1	Every 2 weeks ±1 week ^a^	(every 12 weeks)
Informed consent	X				
Demographics	X				
Medical history	X				
Concomitant illnesses	X				
Concurrent medications	X	X	X	X	
Clinical assessment and physical examination	X	X		X	
Performance status	X	X		X	
Haematology^b^	X	X		X	
Biochemistry^c^	X	X		X	
Pregnancy test	X				
Serum CEA	X			X^d^	
CT of chest/abdomen/pelvis	X			X^e^	X
Assessment for resection	X			X^e^	
Hepatic angiogram^f^		X^g^			
^99m^Tc-MAA lung shunt study^f^		X^g^			
Adverse events	From consent until 28 days after the last dose of protocol chemotherapy				
EQ-5D HRQoL	X			X^h^	
EORTC QLQ-C30 HRQoL	X			X^i^	
Ongoing review of treatment and survival					X

^a^Before each cycle of chemotherapy unless otherwise stated.

^b^Measurement of haemoglobin, platelets, white blood cells, absolute neutrophils and absolute lymphocytes.

^c^Urea, creatinine, liver enzymes, alkaline phosphatase, bilirubin and albumin.

^d^Every 4 weeks.

^e^Every 8 weeks.

^f^Intervention arm only.

^g^7 days ±4 days before SIRT.

^h^EQ-5D questionnaire completed at 3, 6, 12, 24, 36 months, and annually thereafter.

^i^4 weeks and 12 months after starting treatment.

*CEA* = Carcinoembryonic antigen; *CT* = Computed tomography.

### Sample size calculation and statistical considerations

The potential benefit of adding bevacizumab to FOLFOX is approximately a 4-week increase in median PFS [17]. Based on previously reported data, a median PFS time of 8.5 months may be expected with FOLFOX in patients with liver-only or liver-dominant mCRC (i.e., in the control arm); and therefore, with the addition of bevacizumab, median PFS time could be estimated at 9.4 months. Based on previously reported data of SIRT plus hepatic artery chemotherapy (median PFS of 16 months [24]) and SIRT plus first-line 5-FU/LV chemotherapy (median PFS of 18.6 months [22]), a conservative estimation of median PFS in the intervention arm would be 15 months for patients with liver-only metastases and 10 months for patients with liver-dominant metastatic disease. Using these data and assuming a liver-dominant:liver-only ratio of 40:60, a sample size of at least 450 patients for the SIRFLOX study was estimated to detect an increase in the median PFS from 9.4 months to 12.5 months with 80% power and 95% confidence.

Primary and secondary endpoints will be analysed according to the intention-to-treat (ITT) principle. Response rates will be compared between treatment arms using a test of proportions, and time to event endpoints will be compared using the log-rank test. The primary endpoint will be assessed in the ITT population, and additional analyses will also assess endpoints in sub-groups: no bevacizumab versus with bevacizumab; presence versus absence of extra-hepatic metastases; and tumour involvement of the liver ≤25% versus >25%. Exploratory analyses will be performed adjusting for prognostic factors in a multivariate analysis framework.

## Discussion

The SIRFLOX study will assess the efficacy and safety of SIRT in combination with FOLFOX-based systemic chemotherapy as first-line treatment of patients with inoperable liver-only or liver-predominant mCRC. To date, efficacy and safety data for this combination are available in a limited number of patients [23, 26], and for the use of SIRT in chemotherapy-refractory mCRC [27–29]. Although SIRT has been delivered to more than 35,000 patients in over 600 specialist centres worldwide since 2000, such large-scale studies of this treatment have not been feasible before. However, skilled centres are now numerous enough worldwide to enable recruitment of a large pool of patients for these studies.

Some aspects of the treatment arms of the SIRFLOX study are worth further discussion. The prescribed activity of SIR-Spheres was determined by the results of the previous dose-escalation study of SIR-Spheres + FOLFOX4 [23]. Furthermore, in patients randomised to receive SIRT in the SIRFLOX study, the dose of oxaliplatin is reduced to 60 mg/m^2^ for the first three cycles of chemotherapy, and in subsequent cycles is increased to the standard dose of 85 mg/m^2^. One safety concern is that the oxaliplatin in the chemotherapy regimen is a radio-sensitising agent, which when used in combination with external-beam radiation therapy results in hepatotoxicity at doses >60 mg/m^2^ [30–32]. The earlier dose-escalation study of SIR-Spheres + FOLFOX4 also concluded that oxaliplatin at doses >60 mg/m^2^ in the first three cycles of chemotherapy could increase the occurrence of grade 3 and 4 neutropenia [23].

mFOLFOX6 chemotherapy has become widely adopted for the treatment of mCRC, largely because it does not require the day-2 bolus injections of 5-FU that is part of FOLFOX4 and is therefore more convenient. Although previous trials have shown that first-line SIR-Spheres plus chemotherapy can significantly increase time-to-progression and survival in patients with mCRC [22, 24], these used chemotherapy regimens that are now outdated in the management of mCRC. This provides the rationale for choosing mFOLFOX6 in the study. mFOLFOX6 includes oxaliplatin doses of 85 mg/m^2^ rather than the dose of 100 mg/m^2^ used in FOLFOX6 [33] – thus, this will maximise the time that patients can receive protocol chemotherapy before peripheral neuropathy becomes an issue (which necessitates the removal of oxaliplatin) [34].

Patient stratification in SIRFLOX includes the intention to use bevacizumab. The addition of bevacizumab to chemotherapy regimens may enhance survival times and PFS in patients with mCRC [17, 21, 35, 36], but little is known about the potential added benefits of primary chemotherapy + bevacizumab + SIRT. Data on bevacizumab and the other biological agents such as cetuximab and panitumumab for the treatment of liver-only mCRC are limited and sometimes contradictory [37–41], which has made guidance on the optimal first-line treatment strategies difficult and sometimes conflicting [42]. Indeed, despite the initial enthusiasm with targeted biological agents and evidence that they can improve 2-year survival, no gains in 5-year survival have been observed with these agents [43]. The approach of combining systemic chemotherapy ± biologic agents with targeted radiation therapy may provide enhanced benefits in mCRC.

Another strata that will be assessed in these trials is the absence or presence of extra-hepatic metastases. Although published efficacy data on SIRT and FOLFOX chemotherapy are limited, initial data did indicate considerably longer PFS and OS among patients with liver-only disease [26].

In addition to the SIRFLOX study, SIRT in combination with FOLFOX-based systemic chemotherapy as first-line treatment of patients with inoperable liver-only or liver-predominant mCRC is being investigated in the FOXFIRE trial. The studies have almost identical protocols, and FOXFIRE has as a primary endpoint an *a priori* analysis of OS of all patients included in SIRFLOX and FOXFIRE.

A recent consensus statement on systemic cytotoxic and biological therapies for liver metastases from CRC summarised expert recommendations on the management of patients with non-resectable metastatic tumours [44]. In the accompanying editorial [45], Clary et al. identified the need to optimise outcomes of systemic treatment in patients with non-resectable liver metastases as a key question to be addressed. The SIRFLOX study and FOXFIRE trial aim to address this issue in a large patient population and may help provide important new evidence on the use of SIRT early in the therapeutic cascade to enhance PFS and/or OS in this patient population. The use of SIRT with yttrium-90 resin microspheres was not specifically addressed in the consensus statement, presumably due to the paucity of data [44]. Clary et al. highlighted this omission and concluded, “The optimal use of these modalities is undefined and requires further study” [45]. The results from the SIRFLOX study and FOXFIRE trial should further our understanding of SIRT, may help define the optimal use of this treatment modality in mCRC, and may place this treatment option at the forefront of future consensus guidelines.

## Electronic supplementary material

Additional file 1: **Participating centres and ethics committees that have approved the SIRFLOX study.** Table of participating centres ethics committees that have approved the SIRFLOX study. (PDF 183 KB)

## Abbreviations

BSA: Body surface area
CRC: Colorectal cancer
5-FU: 5-Fluorouracil
HRQoL: Health-related quality of life
ITT: Intention-to-treat
LV: Leucovorin
OS: Overall survival
PFS: Progression-free survival
RECIST: Response evaluation criteria in solid tumours
SIRT: Selective internal radiation therapy.
