# Supplementary material for: Selective Internal Radiation Therapy (SIRT) with yttrium-90 resin microspheres plus standard systemic chemotherapy regimen of FOLFOX versus FOLFOX alone as first-line treatment of non-resectable liver metastases from colorectal cancer: the SIRFLOX study
Source: BMC Cancer. 2014 Dec 1;14:897. doi: 10.1186/1471-2407-14-897 (PMC4289171; doi:10.1186/1471-2407-14-897)
Supplement: Supplementary file 1 — Additional file 1: Participating centres and ethics committees that have approved the SIRFLOX study. Table of participating centres ethics committees that have approved the SIRFLOX study. (PDF 183 KB) [file 12885_2014_5083_MOESM1_ESM.pdf]

Participating centre	Ethics committee	Ethics committee town/city and country
Philipps-Universitat Marburg<br>Tagesklinik Hamatologie Onkologie Landshut<br>Hämato-onkologische Gemeinschaftspraxis und Tagesklinik<br>Krebszentrum Ratingen<br>PraxisKooperation Bonn – Euskirchen – Rheinbach<br>Klinikum rechts der Isar der TU Munchen<br>Universitatsklinikum des Saarlandes<br>Klinikum Magdeburg gGmbH<br>Schwerpunktpraxis für Hämatologie und Onkologie Magdeburg<br>MVZ Ingolstadt, Onkologische Praxis Dr Gehbauer/Dr Maywald<br>Klinikum der Universitat Munchen<br>Universitatsklinik Bonn<br>Universitatsklinikum Magdeburg<br>Stadtisches Klinikum Munchen GmbH<br>Evangelische Kliniken Bonn GmbH, Johanniterkrankenhaus Bonn<br>Klinikum der Johann Wolfgang Goethe - Universität Frankfurt am Main<br>Klinikum Karlsruhe, Städtisches Klinikum Karlsruhe gGmbH<br>Kliniken Essen Mitte<br>Charite Berlin<br>Vivantes Klinikum am Urban, Berlin	Ethics Committee of the Medical Faculty of the University of Munich	Munich, Germany
Hopital Europeen Georges Pompidou<br>Centre Eugene Marquis<br>Hopital de l'Archet II, CHU de Nice<br>CHU de Bordeaux<br>CHU de Grenoble & University Hospital Albert Michallon<br>Centre Hospitalier General de Longjumeau	Laboratory of Medical Ethics and Legal Medicine, University Paris Descartes	Paris, France

**Participating centre**	**Ethics committee**	**Ethics committee town/city and country**
Institut Jules Bordet<br>Universiteit Ziekenhuis Gent<br>Universiteit Ziekenhuis Leuven<br>Antwerp University Hospital<br>CHU Sart Tilman<br>AZ Maria Middelares<br>OL Vrouw Ziekenhuis Aalst<br>Hopital de Jolimont<br>Imelda Ziekenhuis<br>AZ Heilige Familie<br>AZ Sint-Maarten	Ethics Committee	Gent, Belgium
University Hospital of Bologna Policlinico S. Orsola Malpighi	Independent Ethics Committee of the University Hospital Policlinico S	Bologna, Italy
Clinica Universitaria de Navarra<br>Hospital de Navarra	Ethics Committee for Clinical Research of Navarra Department of Health	Pamplona, Spain
Wojskowy Instytut Medyczny<br><br>***(Site in Poland initiated but never recruited any patients)***	Bioethics Commission at the Military Institute of Medicine	Szaserów, Poland
Universitätsspital Zürich<br><br>***(Site in Poland initiated but never recruited any patients)***	Cantonal Ethics Committee	Zürich, Switzerland
Sheba Medical Center	Sheba Medical Center Helsinki Committee	Tel Hashomer, Israel
Rabin Medical Center	Helsinki Committee Rabin MC	Petah-Tikva, Israel

**Participating centre**	**Ethics committee**	**Ethics committee town/city and country**
Ramban Medical Center	Rambam Healthcare Campus Helsinki Committee	Haifa, Israel
Tel Aviv Sourasky Medical Center	Tel Aviv Sourasky Medical Center Helsinki Committee	Tel Aviv, Israel
Shaare-Zedek Medical Center	Shaare Zedek Medical Center Jerusalem Ethics (Helsinki) Committee	Jerusalem, Israel
The University of Auckland	Maori Health Research	Auckland, New Zealand
Wellington Hospital	Human Disability Research Ethics Committee	Wellington, New Zealand
The Royal Melbourne Hospital<br>The Western Hospital	Melbourne Health Human Research Ethics Committee	Parkville, Australia
Royal North Shore Hospital	Northern Sydney Local Health District Human Research Ethics Committee	Sydney, Australia
St George Hospital	South Eastern Sydney Local Health District	Kogarah, Australia
Nepean Cancer Care Centre	Nepean Blue Mountains Local Health District Human Research Ethics Committee	Penrith, Australia
St Vincent’s Hospital	St Vincent's Hospital Sydney Human Research Ethics Committee	Sydney, Australia

**Participating centre**	**Ethics committee**	**Ethics committee town/city and country**
Sydney Adventist Hospital	Adventist HealthCare Ltd Human Research Ethics Committee	Wahroonga, Australia
Royal Hobart Hospital	Human Research Ethics Committee (Tasmania) Network	Hobart, Australia
Monash Medical Centre	Monash Health Human Research Ethics Committees	Melbourne, Australia
Peninsula and South Eastern Haematology and Oncology Group	Bellberry Limited	Eastwood, Australia
Maroondah Hospital	Eastern Health Office of Research and Ethics	Box Hill, Australia
Wesley Medical Centre	Unitingcare Queensland Human Research Ethics Committee	Brisbane, Australia
Princess Alexandra Hospital	Metro South Health	Woolloongabba, Australia
Royal Perth Hospital	Royal Perth Hospital Human Research Ethics Committee	Perth, Australia
Sir Charles Gairdner Hospital	Sir Charles Gairdner Group Human Research Ethics Committee	Perth, Australia
Hollywood Private Hospital	Hollywood Private Hospital Research Ethics Committee	Perth, Australia
St John of God Murdoch Hospital	St John of God Health Care Ethics Committee	Perth, Australia

**Participating centre**	**Ethics committee**	**Ethics committee town/city and country**
Mount Medical Centre	Mount Hospital Ethics Committee	Perth, Australia
Royal Adelaide Hospital	Royal Adelaide Hospital Research Ethics Committee	Adelaide, Australia
Queen Elizabeth II Hospital	Queen Elizabeth Hospital Ethics	Woodville South, Australia
Flinders Medical Centre	Flinders Medical Centre Research Ethics	Bedford Park, Australia
Altru Health System	Altru Health System Institutional Review Board	Grand Forks, USA
Froedtert Memorial Lutheran Hospital	Medical College of Wisconsin Froedtert Hospital Institutional Review Board	Milwaukee, USA
Aurora St Luke's Medical Center	Aurora Institutional Review Board	Milwaukee, USA
University of Louisville	The University of Louisville Institutional Review Board	Louisville, USA
University of Maryland	University of Maryland, Baltimore Institutional Review Board	Baltimore, USA
Abbott Northwestern	Allina Hospitals & Clinics Institutional Review Board	Minneapolis, USA

**Participating centre**	**Ethics committee**	**Ethics committee town/city and country**
Florida International University<br>University of Washington<br>Holy Name Hospital<br>City of Hope<br>Pinnacle Oncology Hematology<br>Arizona Center for Cancer Care<br>University of Illinois Chicago	Western Institutional Review Board	Puyallup, USA
Montefiore Medical Center	West Campus IRB (formerly Montefiore Medical Center Institutional Review Board)	Bronx, USA
Carolinas Medical Center	The Institutional Review Board of the Carolinas HealthCare System (pre-2012) and Chesapeake IRB (post 2012)	Charlotte and Columbia, USA [respectively]
Adventist Midwest Health	Adventist Midwest Health Institutional Review Board	La Grange, USA
Ingalls Memorial Hospital	Ingalls Hospital Institutional Review Board	Harvey, USA
St Mark's Hospital	St Mark's Hospital Institutional Review Board	Salt Lake City, USA
William Beaumont Hospital	William Beaumont Hospital Human Investigation Committee	Royal Oak, USA

Participating centre	Ethics committee	Ethics committee town/city and country
West Suburban Medical Center	Vanguard Health Chicago Institutional Review Board	Oak Park, USA
University of Pittsburgh Medical Center	University of Pittsburgh Institutional Review Board	Pittsburgh, USA
